# Biogenic Amine Metabolism and Its Genetic Variations in Autism Spectrum Disorder: A Comprehensive Overview

**DOI:** 10.3390/biom15040539

**Published:** 2025-04-07

**Authors:** Claudio Tabolacci, Angela Caruso, Martina Micai, Giulia Galati, Carla Lintas, Maria Elena Pisanu, Maria Luisa Scattoni

**Affiliations:** 1 Coordination and Promotion of Research, Istituto Superiore di Sanità, Viale Regina Elena 299, 00161 Rome, Italy; angela.caruso@iss.it (A.C.); martina.micai@iss.it (M.M.); giulia.galati@iss.it (G.G.); marialuisa.scattoni@iss.it (M.L.S.); 2 Research Unit of Medical Genetics, Department of Medicine and Surgery, University Campus Bio-Medico of Rome, Via Alvaro del Portillo 21, 00128 Rome, Italy; c.lintas@unicampus.it; 3 Operative Research Unit of Medical Genetics, Fondazione Policlinico Universitario Campus Bio-Medico, Via Alvaro del Portillo 200, 00128 Rome, Italy; 4 Core Facilities, High Resolution NMR Unit, Istituto Superiore di Sanità, 00161 Rome, Italy; mariaelena.pisanu@iss.it

**Keywords:** autism spectrum disorder, biogenic amines, metabolism, neurodevelopmental disorders

## Abstract

Autism spectrum disorder (ASD) is a genetically heterogeneous syndrome characterized by repetitive, restricted, and stereotyped behaviors, along with persistent difficulties with social interaction and communication. Despite its increasing prevalence globally, the underlying pathogenic mechanisms of this complex neurodevelopmental disorder remain poorly understood. Therefore, the identification of reliable biomarkers could play a crucial role in enabling early screening and more precise classification of ASD subtypes, offering valuable insights into its physiopathology and aiding the customization of treatment or early interventions. Biogenic amines, including serotonin, histamine, dopamine, epinephrine, norepinephrine, and polyamines, are a class of organic compounds mainly produced by the decarboxylation of amino acids. A substantial portion of the genetic variation observed in ASD has been linked to genes that are either directly or indirectly involved in the metabolism of biogenic amines. Their potential involvement in ASD has become an area of growing interest due to their pleiotropic activities in the central nervous system, where they act as both neurotransmitters and neuromodulators or hormones. This review examines the role of biogenic amines in ASD, with a particular focus on genetic alterations in the enzymes responsible for their synthesis and degradation.

## 1. Introduction

According to the Diagnostic and Statistical Manual of Mental Disorders, Fifth Edition [1], autism spectrum disorder (ASD) is characterized by persistent deficits in reciprocal social communication and interaction, along with restricted and repetitive patterns of behavior, interests, or activities. These symptoms must manifest during early development, significantly impairing social, occupational, or other important areas of functioning, and cannot be better explained by intellectual disability or global developmental delay. ASD is frequently associated with a wide range of co-occurring psychiatric and medical conditions, varying significantly in severity among individuals. These include other neurodevelopmental disorders (NDDs), including developmental coordination disorder and attention-deficit/hyperactivity disorder (ADHD), as well as sleep–wake disturbances, gastrointestinal issues, anxiety disorders, overweight/obesity, feeding and eating disorders, elimination disorders, disruptive behaviors, somatic symptom and related disorders, depression, and epilepsy [2,3]. The presence of these comorbidities further complicates clinical management [4]. According to the CDC’s Autism and Developmental Disabilities Monitoring (ADDM) Network, approximately 1 in 36 children is diagnosed with ASD, with a prevalence nearly four times higher in boys than in girls [5].

The clinical heterogeneity of ASD is reflected by a high genetic heterogeneity and by the interaction of the genetic background with the environment. For this reason, it is possible to distinguish different forms of “autism”: Mendelian or monogenic autisms associated with a single pathogenetic variants, polygenic autisms in which many gene variants contribute to the final phenotype, and multifactorial forms in which risk polymorphisms interact with adverse environmental factors (for instance, a maternal infection during the first trimester of pregnancy), causing the onset of the disease [6]. Currently, no medications have been clearly proven effective for treating the symptoms of ASD. However, significant interest has emerged in identifying biomarkers that could assist in the screening, diagnosis, and targeted intervention of ASD. Potential markers include neurophysiological measure (e.g., EEG and eye tracking), neuroimaging (e.g., functional MRI), and other physiological measures, as well as genetic, transcriptomic, proteomic, and metabolomic markers [7,8]. The complexity of ASD requires personalized therapeutic approaches that integrate biomarker identification and account for genetic and epigenetic factors, allowing for tailored interventions addressing individual clinical presentations and co-occurring conditions [9]. In this context, to reach a genetic diagnosis may be crucial to design personalized molecular or pharmacological therapies aiming at correcting the “molecular defect.” However, personalized medicine represents a challenge for neurodevelopmental disorders such as ASD. Few successful attempts have been carried out to date [10], and this reflects the complexity of ASD etiology. Hence, more work is needed to strengthen the transferability of genetic information into clinical practice [11].

Although ASD is behaviorally heterogeneous, its biological basis remains largely not understood. The current understanding indicates that the etiology of ASD arises from a complex interplay between genetic, epigenetic, and environmental factors [12]. Several metabolic and neurochemical systems, including monoamines (e.g., serotonin), glutamate/γ-aminobutyric acid (GABA), and neuropeptides, are implicated in the pathophysiology of ASD, as they regulate key processes in central nervous system (CNS) neurodevelopment [8,13,14].

This review explores the central role of biogenic amines (BAs) in ASD. These molecules are involved in several metabolic processes and function as neurotransmitters, hormones, and immune modulators. Specifically, they influence brain activity, behavior, and inflammatory responses, and support normal cell growth and proliferation [15]. The present work focuses on genetic alterations in the genes encoding for enzymes involved in the synthesis and degradation of BAs. Indeed, polymorphisms and rare variants in these genes have been associated with ASD thanks to the advent of pangenomic techniques in the last two decades, including genome-wide association studies, whole-exome sequencing studies, and array–CGH studies [16].

## 2. Classification of Biogenic Amines

BAs are organic low-molecular-weight molecules containing an amino group (-NH₂) that originate from the decarboxylation or deamination of precursor amino acids [17]. They are classified as endogenous and exogenous based on their origin. Endogenous BAs, produced by tissues, include catecholamines (i.e., dopamine, epinephrine, norepinephrine), indolamines (serotonin or 5-hydroxyltryptamine), and imidazolamines (such as histamine). These are particularly important in the nervous system, acting as neurotransmitters to modulate cognitive, emotional, and motor functions [18]. Exogenous BAs, mostly derived from food, are products of amino acid decarboxylation and are not directly involved in neurotransmission. However, they have significant physiological and pathological effects, often influencing local metabolism or inflammation. These include histamine, tryptamine, putrescine (PUT), cadaverine, 2-phenylethylamine, and tyramine [18,19,20].

BAs can also be classified based on their chemical structure, characterized by the presence of one or more amine groups (monoamines, diamines, or polyamines) directly attached to an aliphatic or aromatic carbon atom [21,22]. Monoamines, the most studied class of BAs, are derived from the modification of a single amino acid. They include (i) catecholamines, derived from L-tyrosine (and L-phenylalanine), which affect regulatory functions in CNS and peripheral systems, such as mood, alertness, and stress responses; (ii) indolamines, derived from L-tryptophan, which regulate mood, sleep, appetite, and cognitive functions; (iii) L-histidine-derived imidazolamines, which regulate alertness, immune response, and gastric secretion; and (iv) tyramine, produced by L-tyrosine decarboxylation, which acts as a vasoconstrictor and affects blood pressure [22]. Additionally, octopamine, a derivative of tyrosine, plays a role in neurotransmission and metabolic regulation [23]. Diamines are another group of BAs, characterized by the presence of two amino groups. They include PUT and cadaverine, derived from the decarboxylation of arginine and lysine, respectively. At physiological concentrations, they contribute to cellular homeostasis and growth modulation [24]. Polyamines are a class of BAs characterizing by multiple amino groups in their chemical structure. These molecules are essential for cell maintenance, protein synthesis, and DNA stability. Key polyamines include (i) spermidine (SPD), derived from putrescine, which stabilizes DNA structure, regulates the cell cycle, and influences aging processes and autophagy; (ii) spermine (SPM), a more complex polyamine, which supports membrane structure, cellular fluidity, and chromosome protection during cell division; and (iii) agmatine, derived from arginine, which modulates neurotransmitter activity, regulates blood pressure, and protects against oxidative stress [25].

BAs are also classified as aliphatic, including PUT, cadaverine, agmatine (AGM), SPM, and SPD; aromatic, including tyramine, β-phenylethylamine, octopamine, dopamine, and norepinephrine; and heterocyclic, including serotonin, histamine, and tryptamine [26]. The nomenclature of the main BAs discussed in the present review is reported in Table 1.

The metabolism of BAs is a dynamic and highly regulated process mediated by a complex enzymatic system. These processes are fundamental for maintaining homeostasis and ensuring proper biological function by controlling the synthesis, release, and degradation of BAs [27,28]. BAs are primarily synthesized by the decarboxylation of amino acids, a reaction catalyzed by substrate-specific decarboxylase enzymes [28].

The degradation of BAs is mainly mediated by (i) monoamine oxidases (MAOs), located in nerve terminals and various other cells, which oxidize amines such as dopamine and serotonin into inactive metabolites (aldehydes and hydrogen peroxide); (ii) diamine oxidase (DAO), mainly found in intestinal and renal tissues, which removes amino groups and degrades histamine and tyramine, converting them to inactive compounds; (iii) catechol-O-methyltransferase (COMT), which methylates BAs, thereby inactivating them; and (iv) aldehyde dehydrogenase (ALDH), which converts aldehydes produced during oxidative deamination to carboxylic acids [28,29,30].

**Figure 1:**
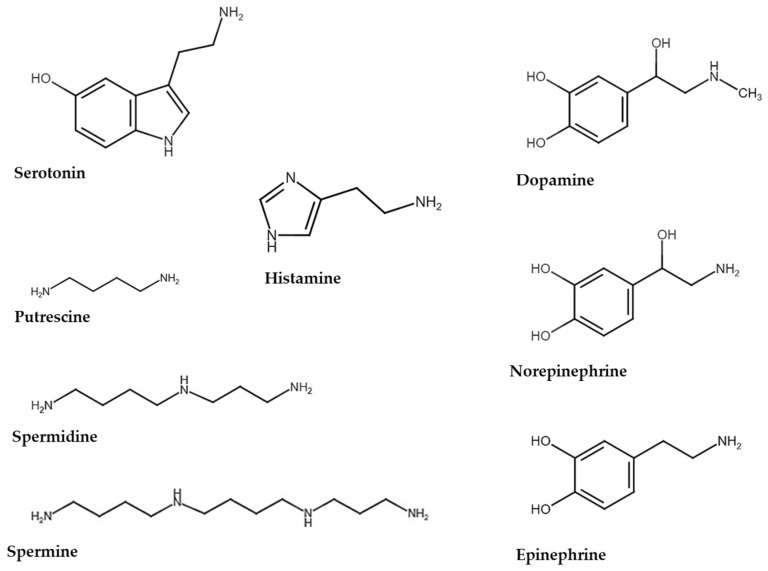
Chemical structure of principal biogenic amines (BAs) discussed in the present review. Chemical structures are drawn using the Chemical Sketch online tool (https://www.rcsb.org/chemical-sketch; Accessed on 7 February 2025).

The balance between the synthesis and degradation of BAs is critical to maintaining appropriate levels in the blood and tissues, preventing excessive receptor activation, and ensuring proper physiological functions. This balance is crucial to avoiding alterations that can affect both brain and systemic functions. The main BAs discussed in this review are summarized in Figure 1.

## 3. Serotonin

The role of serotonin (5-HT) in ASD is a complex and multifactorial issue. Several theories support that dysfunction in the serotonin system—such as synthesis, transport, and metabolism—contributes significantly to the pathophysiology of ASD. Altered serotonin levels during early development may disrupt brain circuitry, potentially leading to the manifestation of autistic symptoms. In the CNS, serotonin modulates behavior, mood, cognition, and homeostasis, influencing processes such as stress response, learning, motor control, and circadian rhythms. In the peripheral system, it controls gastrointestinal motility, cardiovascular functions (e.g., vasoconstriction and platelet aggregation), and hormonal regulation, while also interacting with the gut microbiota to influence immunity, inflammation, and oxidative stress [31].

Indolamine serotonin is a monoamine neurotransmitter synthesized from the essential amino acid L-tryptophan (Trp), which must be obtained exclusively from dietary sources [32]. Serotonin regulates many physiological and behavioral functions, often with diverse and even opposing effects. These functions are mediated by specific serotonin receptors (5-HTRs), which are widely distributed across different tissues. Seven families of 5-HTRs (for 14 subtypes) have been identified. These receptors, excluding the 5-HT3R (ligand-gated ion channel), are G protein-coupled receptors (GPCRs) [33,34].

The biosynthesis of serotonin occurs in two enzymatic steps. First, Trp is hydroxylated by L-tryptophan hydroxylase (TPH), the rate-limiting enzyme, to form 5-hydroxytryptophan (5-HTP). This reaction requires oxygen and tetrahydrobiopterin (BH4) as a cofactor. In the second step, 5-HTP is decarboxylated by aromatic amino acid decarboxylase (AADC), which requires vitamin B6 (pyridoxine) as a cofactor to produce serotonin. TPH exists in two isoforms: TPH-1, predominantly expressed in peripheral tissues like the gastrointestinal tract and pineal gland, and TPH-2, which is selectively expressed in the CNS [32]. Although serotonin cannot cross the blood-brain barrier, its synthesis occurs separately in the brain and peripheral tissues. In the periphery, it is mainly synthesized in intestinal enterochromaffin cells, whereas platelets serve as an important storage site despite their limited ability to synthesize serotonin. Approximately 90–95% of the body’s serotonin is located in platelets, with less than 1% circulating freely in the bloodstream [35]. Once synthesized, serotonin is stored in synaptic vesicles within serotonergic neurons. After exerting its effect, the serotonin transporter (SERT) facilitates the reuptake of serotonin into the presynaptic neuron, allowing it to be either recycled for future release or targeted for degradation [36].

**Figure 2:**
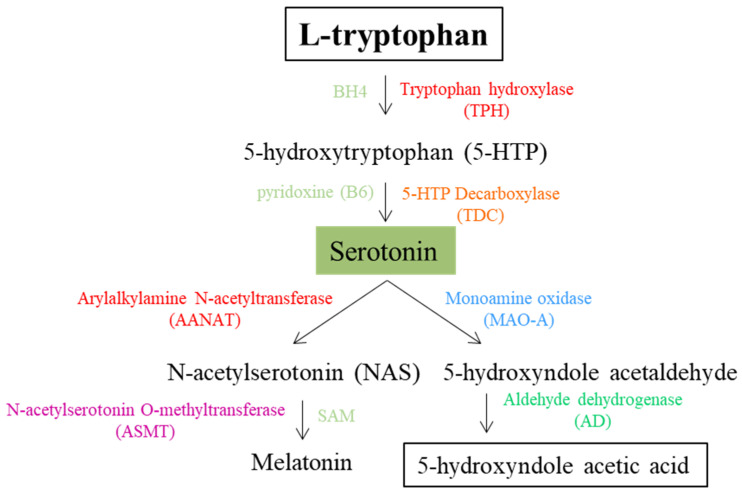
The biochemical pathway of serotonin (5-HT) synthesis and metabolism. L-tryptophan, the essential precursor of 5-HT, is the substrate of the enzyme tryptophan hydroxylase (TPH) that represents the rate-limiting step for 5-HT synthesis. BH4—tetrahydrobiopterin; SAM—S-adenosyl-methionine.

The metabolism of serotonin follows two main pathways: melatonin synthesis and oxidative degradation. In melatonin synthesis, serotonin is converted to N-acetylserotonin (NAS) by arylalkylamine N-acetyltransferase (AANAT). Then, NAS is methylated by N-acetylserotonin O-methyltransferase (ASMT) to form melatonin, a hormone that regulates circadian rhythm [37,38,39]. There are two isoforms of MAO: (i) MAO-A, which is mainly found in the liver, intestine, and CNS, and is responsible for the deamination of serotonin and norepinephrine; and (ii) MAO-B, which is mainly found in the brain and platelets, and is specific to dopamine [40]. Not-recycled serotonin is mainly degraded by MAO-A, which catalyzes the oxidation of serotonin to 5-hydroxyindoleacetaldehyde and then by aldehyde dehydrogenase (ALDH), which further metabolizes 5-hydroxyindoleacetaldehyde to 5-hydroxyindoleacetic acid, an inactive metabolite that is excreted in the urine [37]. The serotonin metabolic pathway is summarized in Figure 2.

One of the most well-known aspects of serotonin in ASD is hyperserotonemia, characterized by elevated serotonin levels in the blood. Although extensively investigated as a potential biological endophenotype of ASD—being both associated with the condition and partially heritable—the exact causes and clinical implications of hyperserotonemia in ASD remain unclear [41,42,43]. A recent systematic review by Esposito and colleagues confirms elevated serotonin levels in individuals with ASD and the consistent positive correlation between serotonin levels and ASD, although the link between hyperserotonemia and other clinical outcomes, such as symptom severity or co-occurring conditions, remains ambiguous [44]. There is no universally accepted approach to defining and measuring hyperserotonemia, which complicates the interpretation of findings. The standardization of analytical techniques, such as the selection of the biological matrix and the sample preparation procedures, remains crucial in minimizing the heterogeneity of findings [44].

Various hypotheses have been proposed to explain the occurrence of hyperserotonemia in ASD. During early development, elevated serotonin in the blood can cross into the fetal brain, where prolonged exposure disrupts physiological serotonin innervation. This disruption continues throughout development, contributing to the onset of ASD symptoms [45]. These hypotheses are supported by animal models of hyperserotonemia, which show a reduction in serotoninergic terminals, alterations in cortical organization, changes in dendritic arborization in target tissues, and autistic-like behavioral traits [46].

One mechanism involves the increased density of the SERT, encoded by the *SLC6A4* gene, in the platelet membrane of individuals with ASD. This may result in enhanced serotonin uptake from the bloodstream [47]. Genetic linkage and association studies suggest a connection to the chromosomal region containing the *SERT* gene [48]. Another potential explanation is dysregulation of the serotonergic system, which may disrupt immune signaling pathways and affect neural-immune interactions, ultimately leading to abnormalities in neuronal connectivity and synaptic function [48]. The genetic variations of genes encoding for serotonin metabolism enzymes related to ASD are shown in Table 2.

The *TPH1* and *TPH2* genes encode the rate-limiting enzymes responsible for serotonin biosynthesis, a process that is notably altered in ASD. Ramoz and colleagues conducted a study on a large cohort of 352 families with autism, including clinically defined subsets with severe obsessive-compulsive behaviors (sOCBs) and self-stimulatory behaviors (SSBs) [49]. Their analysis found no evidence of an association between autism and SNPs or haplotypes of the *TPH1* and *TPH2* genes, either in the full cohort or within the sOCB and SSB subsets [49]. *TPH2* is a recently identified tryptophan hydroxylase gene located on chromosome 12q21.1. Coon and coworkers initially reported a potential association between ASD and *TPH2*, analyzing human sequence variants across 6467 nucleotides, covering all 11 exons of *TPH2*, 248 nucleotides upstream of the start codon, and 935 nucleotides downstream of the stop codon [50]. They identified 18 variants in a cohort of 88 individuals with autism, compared to 95 control subjects [50]. However, three subsequent studies failed to replicate this finding [49,76,77]. More recently, Yang and colleagues reported a significant association between *TPH2* variants and ASD, particularly with repetitive behavioral patterns [78]. Additionally, Singh and colleagues found a positive association between *TPH2* haplotypes and ASD in the Indian population, noting that the interaction between *ITGB3* and *TPH2* markers increases the risk of ASD [79]. Yu et al. (2002) found no significant association between single-nucleotide polymorphisms in the *DDC* gene and ASD [80]. In contrast, Toma and coworkers reported that, in a case-control study of 326 unrelated autistic individuals and 350 gender-matched controls from Spain, common allelic variants in the *DDC* gene may contribute to autism susceptibility [51].

As previously stated, MAO is the enzyme responsible for the degradation of serotonin to 5-hydroxy-3-indoleacetaldehyde (5-HIAL). Elevated levels of 5-hydroxy-3-indoleacetacetic acid (5-HIAA) have been found in the urine of hyperserotonemic autistic subjects. Several studies suggest that variations in the *MAOA*-type gene (carried by the X chromosome), particularly certain polymorphisms and haplotypes, are associated with an increased risk and severity of ASD, with notable sex-specific effects. Specifically, the MAOA marker rs6323 has been linked to ASD, with the low-activity T allele of rs6323 increasing the risk, especially in male probands. The observed pattern of linkage disequilibrium strengthens the hypothesis of a sex-specific role for *MAOA* in the pathophysiology of ASD [58]. These findings suggest that the genetic influence of *MAOA* may vary by sex, offering valuable insights into the complex mechanisms underlying ASD. In a case-control association study, male children carrying four tandem repeats in the promoter region of the *MAOA* gene were found to have a twofold higher risk of developing autism compared to those carrying the 3-repeat allele [81]. A study examining the association between autism severity and a functional polymorphism in the *MAOA* promoter region found that boys with the low-activity 3-repeat *MAOA* allele exhibited more severe sensory behaviors, greater difficulties with arousal regulation, higher levels of aggression, and poorer social communication skills compared to males with the high-activity allele [61]. Although serotonin is preferentially oxidized by MAO-A, several polymorphisms and haplotypes in the *MAOB* gene (adjacent to *MAOA* in X chromosome) are associated with an increased severity of autistic symptoms and higher platelet serotonin content mainly in the male population [82].

Serotonin has been identified as a precursor of melatonin. Since the conversion of serotonin to melatonin relies on specific enzymes, such as AANAT and ASMT, any dysfunctions in these enzymes could influence both serotonin and melatonin levels, potentially affecting behavior and the regulation of biological rhythms. Disruptions in these pathways might contribute to a variety of neurological and behavioral symptoms. Preliminary studies have suggested that genetic variations or mutations in the genes encoding these enzymes could be implicated in certain forms of autism. It has been reported that autistic individuals show reduced activity of both AANAT and ASMT enzymes, which are crucial for melatonin synthesis. This reduction in enzyme function may help explain the lower levels of melatonin observed in autism [83,84]. Interestingly, although ASMT has been phenotypically correlated with melatonin, its heritability differs, especially in autistic children, with high heritability observed for ASMT and low heritability for melatonin [85]. Specifically, two polymorphisms located in the promoter (rs4446909 and rs5989681) are more prevalent in autistic individuals compared to controls and are linked to a significant reduction in ASMT transcripts in blood cell lines [72]. In a nationally representative twin cohort study from Sweden, a symptom-based analysis aimed at exploring potential associations between ASMT and autistic-like traits in the general population identified a nominally significant link in girls between a single-nucleotide polymorphism (rs5949028) in the final intron of ASMT and impairments in social interaction. However, no significant associations were found with traits related to language impairment or restricted and repetitive behaviors [73]. *ASMT* genotypes have been shown to influence sleep and circadian rhythms in adults with autism [86]. These studies suggest that genetic variations in the *AANAT* and *ASMT* genes, which are involved in melatonin synthesis, are associated with low melatonin levels and may contribute to autism spectrum disorders by affecting sleep patterns and social interaction impairments. Since sleep disturbances are among the most common comorbid conditions reported in ASD, understanding the underlying causes of poor sleep in these individuals is crucial for improving treatment options, which remain a clinical priority.

In addition to its role as a neurotransmitter, serotonin acts as a crucial trophic factor, regulating neuronal growth, differentiation, migration, and survival during neurodevelopment. The trophic function begins before synapse formation and continues through prenatal and early postnatal development. Serotonin aids in neuronal development, and its receptors also regulate key processes such as cell division, differentiation (e.g., neurogenesis, dendrite formation, synaptogenesis), axon branching, neuronal migration, and apoptosis. Even brief disturbances in the serotonin system during development can result in long-term changes in brain function and behavior [87], suggesting that early alterations in serotonin regulation contribute to the emergence and pathophysiology of ASD. Moreover, in utero exposure to substances that elevate blood serotonin levels, including selective serotonin reuptake inhibitors (SSRIs), valproic acid, alcohol, and cocaine, has been associated with increased rates of abnormal embryogenesis and a higher risk of ASD [48,88,89]. Interestingly, peripheral serotonin can still affect the brain function in autism subjects through indirect pathways. In fact, this biogenic amine might influence other neurotransmitter synthesis, immune function, and the gut-brain axis, potentially affecting mood, sensory processing, and behavior. Although peripheral serotonin cannot directly cross the blood-brain barrier, its dysregulation may contribute to issues like gastrointestinal distress, sensory sensitivities, and emotional regulation, all of which are commonly observed in ASD [90,91]. In summary, while elevated serotonin levels are a robust biomarker in ASD, the clinical implication of hyperserotonemia remains uncertain. Further research is needed to clarify the exact role of serotonin in the neurobiological underpinnings of ASD, refine measurement techniques, and assess its potential as a therapeutic target.

## 4. Histamine

Histamine (β-imidazolylethylamine) is one of the most important players in several physiological and pathological processes, including immune regulatory functions, controlling gastrointestinal secretion, neurotransmission in the CNS, and vascular permeability [92]. The main sources of histamine are mast cells (MCs) and basophils, where this amine is stored in specific cytoplasmic granules. In the brain, histamine-releasing neurons are exclusively found in the tuberomammillary nucleus (TMN) of the hypothalamus. This central histamine system regulates several CNS functions, including circadian rhythms, the control of pituitary hormone release, appetite, and water retention [93]. Moreover, histamine shows neuromodulator activity in the regulation of fetal brain development [94,95].

The effects of histamine are due to its interaction with four GPCRs, encoded by the genes histamine receptor H1 (*HRH1*), *HRH2*, *HRH3*, and *HRH4*. In detail, H1 is a post-synaptic receptor mainly expressed in the brain, whereas H2 is a post-synaptic receptor mainly involved in gastric acid secretion. The pre-synaptic receptor H3 is expressed in the CNS and is involved in the inhibition of histamine release. The post-synaptic receptor H4 is predominantly expressed in immune cells [96,97,98,99,100]. Histamine, along with the expression and distribution of its receptors (HRs), is closely associated with several allergic diseases, such as allergic rhinitis and asthma, and atopic dermatitis [91], regulating cytokine production by immune cells in allergic inflammation [101]. In this regard, several studies suggest a possible link between allergy and NDDs, including ASD [102]. A possible relationship between allergy-mediated MC activation and ASD has been hypothesized, supported by evidence that neuroinflammation-related cytokines/chemokines, such as interleukine-1β (IL-1β), IL-8 (CXCL8), and tumor necrosis factor α (TNF-α), are widely correlated with the development of ASD [103].

**Figure 3:**
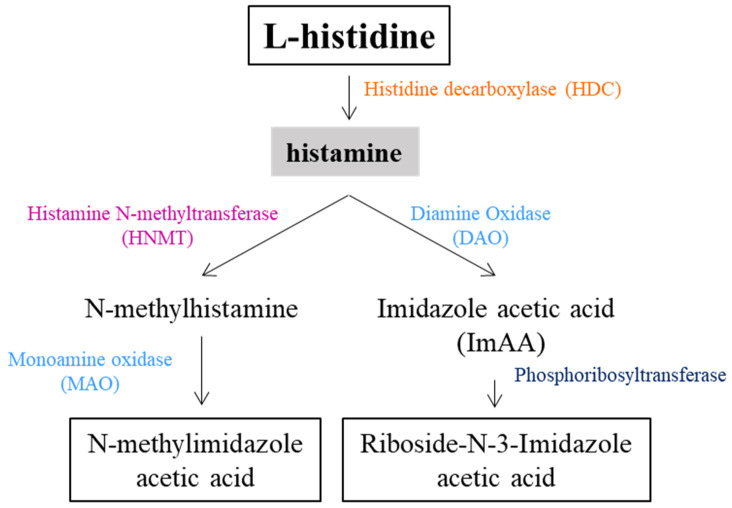
Schematic presentation of histamine synthesis and degradation.

Histamine, a heterocyclic monoamine consisting of an imidazole ring with an ethylamine side chain, is derived from L-histidine (His) and is synthesized in a one-step process in which His is decarboxylated by a reaction catalyzed by L-histidine decarboxylase (HDC) (Figure 3).

In mammals, histamine synthesis is closely linked to HDC activity and the presence of MCs, and is tightly regulated by precursor availability and enzyme activity. In particular, histamine is rapidly metabolized by two primary enzymatic pathways [104]: Histamine-N-methyltransferase (HMT), which converts histamine to N-methylhistamine, mainly in the CNS, intestinal smooth muscle, liver, kidney, and small intestine mucosa; and DAO, which degrades the remaining histamine to imidazole acetic acid (ImAA), with high activity in the small intestine, liver, kidney, eosinophils, placenta, and skin. After metabolism, histamine is excreted in the urine as imidazole-4(5)-acetic acid (ImAA) and its conjugates, such as riboside-N-3-imidazole acetic acid [105].

The genetic variations of genes encoding for histamine metabolism enzymes related to ASD are shown in Table 3.

As previously stated, histamine is synthetized by decarboxylation of L-histidine through the activity of HDC, the regulation of which appears to be important for the physiological and pathological role of histamine itself [100]. A functional mutation in *HDC* has been associated with Tourette syndrome (TS), a neuropsychiatric disorder characterized by motor and vocal tics that represents one of the most frequent comorbidities of ASD, with which it shares similar genetic risk factors [106,107]. Indeed, Ercan-Sencicek and colleagues described, in a family study in which TS is transmitted in a Mendelian manner, a nonsense mutation in *HDC* (G-to-A transition located in exon 9 with consequent premature termination signal) [106]. Moreover, the involvement of the histaminergic system in TS has also been demonstrated in large population-based studies [108,109]. Recently, a 15q21.2 deletion encompassing a genomic region of approximately 175 Kb was reported, which involved the entire *HDC* gene in an individual with borderline intellectual disability with autistic traits [110].

Histamine homeostasis is closely regulated at the catabolic level through the activity of HMT (encoding gene *HNMT*; mainly expressed in the CNS) and DAO (encoding gene *AOC1*; mainly expressed in the digestive tract) [111]. It has been demonstrated that the *HNMT*, *HRH1*, *HRH2*, and *HRH3* genes are differentially expressed in autistic individuals [107], although the directionality of these variations is not always consistent. Mulatinho and co-workers presented an individual with severe intellectual disability, ASD traits, congenital malformations (hypospadia and omphalocele), and episodes of high blood pressure, with an interstitial deletion at 2q22.1q22.3 (~6 Mb) encompassing eight genes, including *HNMT* [112]. More recently, a nonsense mutation in the first exon of the *HNMT* gene (C-to-T transition) leading to a premature stop codon is described in an autistic patient with sleep disorders, delayed speech, and gastrointestinal problems [113]. Although some variants of *DAO* are related to ADHD [114], to the best of our knowledge, only one report shows a possible correlation of an interstitial deletion of the long arm of chromosome 7, which includes DAO, among others, and autistic behavioral symptoms [115]. The expression and distribution of *MAO* isoforms differ markedly in both embryonic development and adult tissues [116]. In particular, MAO-B dysfunctions have been associated with several pathological conditions, including depression and neurodegenerative diseases [82,116].

**Table 3:** Enzymes of histamine metabolism and relative encoding genes. The association with autism spectrum disorder (ASD) is reported.

Enzyme Symbol	Name	Protein ID	E.C. Number	Gene Name	Gene ID	OMIM Gene ID	Disease Association (MIM Phenotype)	Gene Association with ASD (SFARI and Pubmed)	ASD or ASD Associated NDD SNV or CNV	Type of Study	Ref.
HDC	L-histidine decarboxylase	P19113	4.1.1.22	*HDC*	3067	*142704	Gilles de la Tourette syndrome (#137580)	Not listed in SFARI database; found on PubMed in association with autism	CNV, del15q21.2	Array-CGH	[110]
DAO	Diamine oxidase; amine oxidase copper-containing 1	P19801	1.4.3.22	*AOC1*	26	*104610	Not correlated with a MIM phenototype	Not listed in SFARI database; found on PubMed in association with autism	CNV, del7q35q36.1	Array-CGH	[115]
HMT	Histamine N-methyltransferase	P50135	2.1.1.8	*HNMT*	3176	*605238	Susceptibility to asthma (#600807) and intellectual developmental disorder, autosomal recessive 51—MRT51 (#616739)	Not listed in SFARI database; found on PubMed in association with autism	c.88 C > T (p.Glu30Ter)	WES	[113]
MAO (MAO-B)	Monoamine oxidase (2 isoforms)	P27338	1.4.3.4	*MAOB*	4129	*309860	Not correlated with a MIM phenototype	Strong candidate gene	rs1799836 correlated with serotonin levels in autism	Genotyping	[82]
									rs6324 correlated with ASD symptom severity	Genotyping	[82]
									CNV, delchrXq11.3q11.3	Array-CGH	[62]
									CNV, delchrXp11.3-p11.4	Array-CGH	[63]
									c.392G > T (p.Ser131Ile)	WES	[117]

Histamine plays a key role in the brain as a neuromodulator and neurotransmitter, influencing neuroinflammation and microglial functions. Given its involvement in these processes, it is clear that histamine, through its metabolic dynamic and receptor expression, may be involved in the pathophysiology of NDDs, including ASD [107]. Therefore, histamine has been proposed as possible biomarker for ASD, with studies showing significantly higher plasma levels of this BA in children with ASD compared to a control group [118]. However, this biological correlation remains to be further investigated, since plasma histamine levels (produced by neuronal and/or non-neuronal cells) may depend on multiple factors, including the inflammatory state [95].

## 5. Catecholamines

Catecholamines, which include dopamine, epinephrine, and norepinephrine (or noradrenaline; 3,4,β-trihydroxyphenethylamine) are a class of BAs that act as both neurotransmitters when synthetized in sympathetic nerves and hormones when synthesized in the adrenal medulla and released in the bloodstream [119]. Sympathetic neurons and chromaffin cells in the adrenal glands originate from common progenitors in the neural crest, showing similar molecular and biochemical pathways. Catecholamines share a 1,2-benzenediol (catechol) nucleus and are synthesized from L-tyrosine (and L-phenylalanine) [120]. Specifically, dopamine, chemically identified as 4-(2-aminoethyl)-1,2-phenol, is a major monoamine neurotransmitter [121]. L-tyrosin is first hydroxylated to L-DOPA (3,4-dihydroxyphenylalanine) by the tyrosine hydroxylase (TH), the rate-limiting step in dopamine synthesis. L-DOPA is then decarboxylated to dopamine by the DOPA decarboxylase (also known as aromatic L-amino acid decarboxylase; AADC) [122]. Dopamine can be further converted to norepinephrine by the action of dopamine β-hydroxylase (DBH) and, finally, to epinephrine by phenylethanolamine N-methyltransferase (PNMT), which methylates norepinephrine [122].

The metabolism of dopamine is crucial for regulating its action and occurs via two main pathways: oxidative deamination mediated by MAO, and O-methylation mediated by COMT [123,124,125]. MAO is a mitochondrial enzyme that catalyzes the first step of oxidative deamination in dopamine metabolism. MAO-B deaminates dopamine to form 3,4-dihydroxyphenylacetaldehyde (DOPAL), a highly reactive and toxic intermediate metabolite that can induce oxidative stress when accumulated. DOPAL can be further metabolized by two main pathways: ALDH, which converts DOPAL to 3,4-dihydroxyphenylacetic acid (DOPAC), and aldehyde reductase, which reduces DOPAL to 3,4-dihydroxyphenylethanol (DOPET), a less toxic product [126].

In the prefrontal cortex, COMT catalyzes the conversion of DOPAC to homovanillic acid (HVA) by adding a methyl group (-CH₃) to the 3-carbon of the benzene ring, producing the major metabolite of dopamine in the CNS and another secondary metabolite, 3-methoxytyramine, which neutralizes their biological activity and prepares them for elimination in urine [127]. COMT is distributed across various tissues, including the brain, liver, and kidneys, and exists in two main forms: soluble and membrane-bound [127].

**Figure 4:**
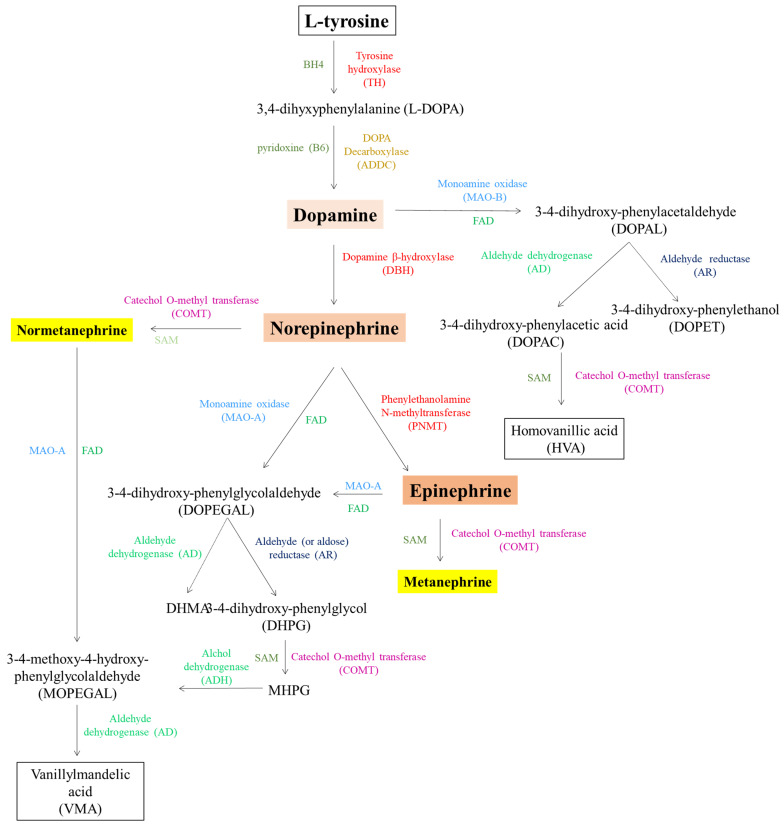
The biochemical pathway of catecholamine synthesis and metabolism. FAD—flavin adenine dinucleotide; BH4—tetrahydrobiopterin; SAM—S-adenosyl-methionine.

The metabolic pathways for norepinephrine and epinephrine are closely related to those for dopamine, mainly involving oxidative deamination with subsequent metabolic reduction and oxidation [40]. Norepinephrine undergoes oxidative deamination by the enzyme MAO-A, which converts it to 3,4-dihydroxyphenylglycolaldehyde (DOPEGAL). This process removes the amine group and transforms the molecule to a more oxidized form [126]. Epinephrine undergoes a similar deamination process, catalyzed by MAO-A, resulting in a compound similar to DOPEGAL, with differences due to the presence of a methyl group on the nitrogen atom in epinephrine [40]. Once DOPEGAL is formed, it undergoes further metabolic modifications via reduction and oxidation pathways. In the reduction process, the enzyme aldehyde reductase, which is present in sympathetic neurons and adrenal chromaffin cells, reduces DOPEGAL to 3,4-dihydroxyphenylglycol (DHPG), a more stable and less reactive compound than its aldehyde precursor [126]. In addition, aldose reductase can also reduce catecholaldehydes (e.g., DOPEGAL) to their corresponding alcohols (e.g., DHPG) more efficiently than aldehyde reductase. In the oxidation process, aldehyde dehydrogenase oxidizes DOPEGAL to vanillylmandelic acid (VMA), which is the major end-product of norepinephrine and epinephrine metabolism. VMA is excreted in the urine and serves as an important marker of adrenergic activity and the body’s stress response [128,129]. COMT, which is active in the sympathetic nervous system, also contributes to the metabolism of norepinephrine and epinephrine. The primary metabolites of COMT’s methylation include metanephrine for epinephrine and normetanephrine for norepinephrine [127]. The metabolic pathway of catecholamines is summarized in Figure 4.

As a key neurotransmitter in mammals’ brains, dopamine is predominantly synthetized in the substantia nigra and in the ventral tegmental area, where it is stored in vesicles by vesicular monoamine transporter 2 (VMAT2, SLC18A2). This transporter, together with VMTA1 (*SLC18A1*), is responsible for the packaging of other monoamines, including histamine, serotonin, and norepinephrine [130]. Dopamine interacts with some GPCRs divided into two subtypes, namely the D1-type family (D1 and D5 receptors) and the D2-type family (D2, D3, and D4 receptors), with opposite effects [131]. These receptors, depending also on their location, mediate the different effects of dopamine, including the regulation of motor functions, hypertension, sleep, memory, cognition, and neuroendocrine secretion [132,133]. Dopamine receptors are also involved in neuroinflammation, which is identified as facilitator of several brain pathologies, including ASD [134,135]. Furthermore, dopamine plays an important role in CNS development, because it regulates the proliferation and migration of neural precursor cells, neuronal differentiation, neurogenesis, and synaptogenesis [136]. It is therefore clear how serotonin signaling must be finely regulated, as its disruption can have long-term physiological and behavioral effects on brain functions [137]. Spatial and temporal regulation of dopamine activity also depends to its two principal clearance mechanisms: (i) reuptake by the dopamine transporter (DAT) that is prominent in striatum, (ii) degradation by the COMT that predominates in the prefrontal cortex [138].

It is interesting to note that neuromodulators do not always act independently. In fact, dopamine and norepinephrine share several overlapping features [139]. In particular, in the CNS, norepinephrine is mainly produced in the locus coeruleus, while epinephrine plays only a small role as neurotransmitter [140]. However, both epinephrine and norepinephrine act on adrenergic receptors, a heterogeneous family of GPCRs divided into three subfamilies (α1, α2, and β receptors) that show different signaling pathways and opposite effects in the body and are predominantly involved in mediating sympathetic activation of peripheral organs and the CNS [141,142,143]. Adrenergic receptors are largely expressed in the CNS and regulate neuronal functions, including synaptic plasticity. An aberrant modulation of this neurotransmitter system may affect neurodevelopment. For instance, polymorphism in the β2 receptor genes (*ADRB2*) may be related to an increased risk of ASD [144]. However, we focused our attention on genetic variations of genes involved in the catecholamine metabolic pathway (Table 4).

Dysfunctions of the catecholaminergic system, due to its neuromodulatory role in a wide range of CNS functions, have often been considered potential contributors to NDDs, including ADHD and ASD [150]. However, the assessment of catecholamine and their metabolite (HVA, VMA, DOPAC, MHPG) levels in the plasma or urine of autistic individuals with respect to control groups has led to contrasting results [151,152,153,154,155].

As previously stated, TH, belonging to the tetrahydrobiopterin-dependent aromatic amino acid hydroxylase family, which also includes phenylalanine hydroxylase and the tryptophan hydroxylases TPH1 and TPH2, is the rate-limiting enzyme of catecholamine biosynthesis. The fact that targeted inactivation of TH results in gestational lethality in mice underlines the key role of this enzyme [57]. A human tyrosine hydroxylase deficiency (THD; OMIM 191290) has been described in patients with *TH* mutations as an autosomal recessive neurometabolic disorder [156]. Interestingly, Reyes and colleagues recently described a young person with THD (heterozygous for two variants in *TH* gene) and ASD as a comorbidity [157].

Several studies underline the strong relationship between COMT, the key enzyme of catecholamine degradation, and neuropsychiatric disorders [158]. In particular, the *COMT* gene has several polymorphisms, although the best characterized one (rs4680) is a substitution of a valine (Val) for a methionine (Met) residue at position 108 (for the cytosolic soluble COMT isoform) or at position 158 (for the membrane-bound COMT isoform) that leads to lower enzymatic activity. This single-nucleotide polymorphism (SNP) is associated with schizophrenia and bipolar disorder [159,160], and some association with ADHD has also been postulated [161]. The potential impact of different COMT allelic variants (rs8192488, rs4680, and rs4818) on autistic individuals has been deeply investigated by Esmaiel and colleagues [145]. In fact, it has been confirmed that the rs4680 variant led to a decrease in the mRNA thermodynamic stability of the COMT (and consequently, its activity), and that this decrease is greater when there is homozygosity for the COMT Met (MM) allele (more common in ASD individuals) compared to homozygosity for the Val (VV) allele [145].

The 22q11.2 deletion syndrome (22q11.2DS) is characterized by a hemizygous deletion in chromosome 22 (~3 Mb) that encompasses the COMT gene, among others. 22q11.2DS is frequently associated with a large variety of symptoms, including neuropsychiatric manifestations, in particular, schizophrenia, ADHD, and ASD [162,163]. In 22q11.2DS individuals, the presents of COMT variants may amplify the effects of COMT haploinsufficiency-related dopaminergic dysfunction [164,165]. In line with these observations, Hidding and co-workers underlined that, in 22q11.2DS individuals, there exists a tight association of the COMT^158^ genotype and autism severity symptoms [147].

Another important enzyme in the catecholaminergic system is dopamine DBH, which is responsible for the conversion of dopamine to norepinephrine, the coding gene of which (*DBH*) appears to be implicated in mood disorders, including major depressive disorder and bipolar disorder [166]. Moreover, several lines of evidence suggest that *DBH* polymorphisms, affecting enzymatic activity, may be related to ASD [148,149,167]

## 6. Diamines and Polyamines

The diamines putrescine (1,4-diaminobutane; PUT) and cadaverine (1,5-diaminopentane; CAD) are often included with the polyamine group and are found in a wide range of animal, plant, and microbial tissues. Derived from the decarboxylation of amino acids such as L-ornithine, L-arginine, and L-methionine, they are essential for many biological functions [168]. Diamines are synthesized by multi-step enzymatic pathways: PUT is produced by the decarboxylation of L-ornithine, an amino acid involved in the urea cycle derived from arginine degradation and catalyzed by the enzyme ornithine decarboxylase (ODC), a pyridoxal-5′-phosphate (PLP)-dependent decarboxylase [169]. PUT is also a precursor for the synthesis of polyamines such as spermidine (SPD) and spermine (SPM) [170]. CAD is formed often by microbial activity as a degradation product of amino acids during the decomposition of organic tissues, generally under anaerobic conditions [171].

Polyamines are polycationic alkylamines, ubiquitously found in all organisms, and are implicated in several biological processes, including cell growth and differentiation, gene regulation, and apoptosis [172,173]. In fact, their protonated amino groups at physiological pH enable them to interact with negatively charged macromolecules such as DNA, RNA, proteins rich in glutamic and aspartic acids, and phospholipids, stabilizing the molecular structures and thus carrying out their biological action. Therefore, polyamine levels are closely regulated by several mechanisms, as they are involved in promoting cell cycle progression [174]. The distribution of these positively charged aliphatic amines in the CNS has attracted considerable interest, since several studies underline their possible role in neuroprotection and age-associated neurological diseases [175]. The primary polyamines include spermidine (N′-(3-aminopropyl)butane-1,4-diamine; SPD), spermine (N,N′-bis(3-aminopropyl)butane-1,4-diamine; SPM), and agmatine (2-(4-aminobutyl)guanidine; AGM) [176,177]. SPM, the most complex polyamine, acts as a scavenger of reactive oxygen species (ROS), reducing oxidative stress. In addition, SPM modulates potassium channels and acts on glutamate receptors (N-methyl-D-aspartate receptor; NMDAR), processes that are integral to learning and memory. In addition to these roles, it provides structural support to cell membranes, contributing to their stability and fluidity, and plays a protective role during cell division by maintaining chromosome integrity [26,170].

**Figure 5:**
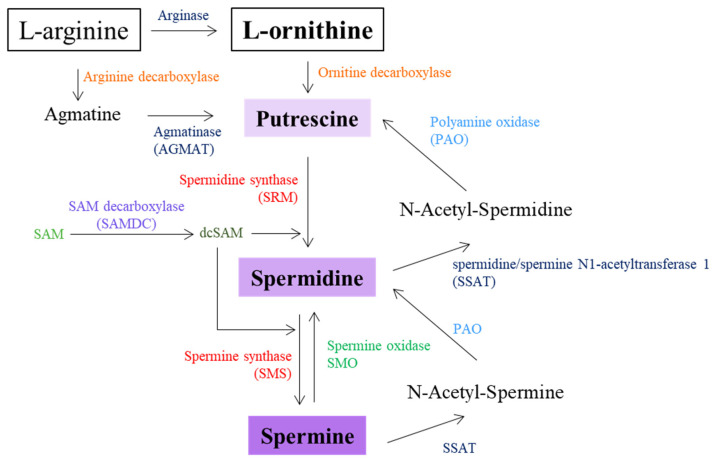
Schematic representation of diamine and polyamine metabolism. SAM—S-adenosyl-methionine; dcSAM—decarboxylated S-adenosylmethionine.

Polyamines are synthesized from amino acids, with ornithine acting as the central precursor. Ornithine decarboxylase (ODC), a key enzyme with rapid turnover, catalyzes the rate-limiting step, converting ornithine to PUT [178,179]. AGM, derived from arginine decarboxylation, also contributes to PUT synthesis via agmatinase (AGMAT) [179,180]. SPD is then formed by the addition of an aminopropyl group provided by decarboxylated S-adenosylmethionine (SAM) to PUT, a reaction catalyzed by spermidine synthase (SRM), while SPM is then synthesized from SPD by spermine synthase (SMS) [181]. Polyamines are catabolized by oxidative pathways involving enzymes such as polyamine oxidases (PAOs), which degrade acetylated polyamines, producing hydrogen peroxide and reactive aldehydes and spermine oxidase (SMO), which converts SPM to SPD, producing hydrogen peroxide and reactive aldehydes. Acetylation by spermidine/spermine N1-acetyl-CoA transferase (SSAT) facilitates subsequent oxidation, ensuring controlled polyamine turnover and preventing toxicity [182,183]. Polyamine metabolism is summarized in Figure 5.

Polyamine homeostasis is tightly regulated by biosynthesis, degradation, transport, and feedback mechanisms [184,185,186,187]. Elevated polyamine levels inhibit ODC and SAMDC (S-adenosylmethionine decarboxylase), preventing excessive biosynthesis that could disrupt normal cellular processes through feedback inhibition. Antizymes that prevent ODC dimerization also promote its degradation by the proteasome [188]. Specialized transport proteins regulate the uptake and efflux of polyamines to maintain intracellular and extracellular balance. Vesicular polyamine transporters (VPATs), identified in astrocytes and mast cells, facilitate the distribution of polyamines between cellular compartments [189]. Finally, the coupling of polyamine metabolism to energy metabolism via acetyl-CoA/CoA recycling participates in the limiting reaction for polyamine degradation (SSAT), connecting it to lipid metabolism and cellular energetics. The genetic variations of genes encoding for polyamine metabolism enzymes related to ASD are shown in Table 5.

As previously stated, ODC (encoding gene *ODC1*) is the rate-limiting enzyme in polyamine biosynthesis, catalyzing the decarboxylation of ornithine to PUT. Is interesting to note that ODC expression is elevated in the CNS, but its antizymes negatively regulate its activity [191], and the abnormal presence of the ODC in the brain is mostly associated with neurodegenerative disorders (e.g., Alzheimer’s disease) [192]. However, it has been described in Bachmann-Bupp syndrome (BABS), a neurodevelopmental disorder associated with developmental delay, hypotonia, and hair loss (OMIM #619075), which is caused by 3′-end mutations of the *ODC1* gene. These mutations produce a truncated form of ODC that evades proteasomal degradation, leading to an accumulation of ODC that, however, maintains its enzymatic activity [193]. The use of difluoromethylornithine (DFMO), a well-known ODC inhibitor, has been shown to improve the behavior of BABS patients [192,193], corroborating evidence that underlines the association between the *ODC1* gain-of-function variants and NDDs [190,194].

Polyamine deficiency has been described in Snyder-Robinson syndrome (SRS; OMIM: 309583), an X-linked intellectual disability condition associated with mutations in the *SMS* gene, resulting in an altered intracellular SPD/SPM ratio [195,196]. Moreover, SMS has been considered a risk gene in a cohort of families with ASD from Saudi Arabia [117]. Although the enzymatic activity of spermidine/spermine N1-acetyl transferase-like 1 remains to be clarified, mutation in the encoding gene (*SATL1*) has been associated with the ASD phenotype [197].

Polyamine imbalances, particularly those involving AGM, are associated with depression, anxiety, and schizophrenia and represent potential therapeutic targets [198]. In particular, AGM acts as a neurotransmitter that exerts inhibitory effects on some receptors, including NMDAR [199]. It has been reported that in ASD individuals, plasma levels of *AGM* are lower with respect to the control group, suggesting that this polyamine might be involved in ASD pathogenesis [200].

## 7. Discussion

The role of BAs in ASD has become an area of growing interest due to their significant implications in both understanding the neurobiological mechanisms underlying the disorder and informing diagnostic and therapeutic strategies. Despite the absence of effective pharmacological treatments targeting the core symptoms of ASD, ongoing research into reliable biomarkers continues to show promise, particularly within the fields of genetics, transcriptomics, proteomics, and metabolomics. Among these, blood serotonin levels have gained particular attention as a potential biomarker for ASD, with the hope that it could eventually facilitate early screening and more precise stratification of ASD subtypes. Nevertheless, the current lack of standardized methodologies for measuring BAs limits their immediate clinical utility [201]. This challenge highlights the critical need for continued research to refine these diagnostic tools and biomarkers.

Although the molecular basis underlying ASD is not yet completely understood, plenty of progress has been made during the last few years. In this regard, large-scale whole-exome sequencing has led to the discovery of many causal ASD genes: many new ASD monogenic forms have been identified [202]. These emerging molecular studies represent a starting point to developing personalized targeted treatment, and a few personalized molecular treatments have already been proposed [203]. Similarly, some research on ASD biomarker identification has given encouraging results: in this regard, hyperserotonemia seems to be a reliable biomarker of ASD [44]. Selective serotonin reuptake inhibitors (SSRIs) are used for the treatment of several ASD-related conditions (i.e., depression, anxiety, and obsessive-compulsive behaviors). Some clinical studies have been conducted on these drugs (e.g., fluoxetine, fluvoxamine, and citalopram), although they have not always led to significant results [204]. On the contrary, the use of SSRIs during pregnancy might be associated with a higher risk of ASD [205,206,207]. Future clinical trials should focus on restoring the “molecular defects” in the biogenic amine metabolism by using agonist or antagonist drugs according to the gene mutations (loss-of-function or gain-of-function mutations). In this regard, methylphenidate is successfully used in the management of ADHD. This drug is involved in dopamine metabolism, as it is a reuptake inhibitor, thereby increasing the presence of this neurotransmitter in the extraneuronal space and prolonging its action. Many studies have shown that the genetic and epigenetic status of the dopamine transporter gene significantly influences the response to methylphenidate [208].

Considering the heterogeneous nature of ASD, a multidimensional approach that integrates genetic, epigenetic, biochemical, and clinical data is essential for advancing our understanding of the disorder. This integrated framework would be instrumental in developing personalized therapeutic strategies better suited to addressing the complexity and variability of ASD’s manifestations. In fact, despite many promising findings from genomics [209], metabolomics [210,211,212], and proteomics [213,214], studies on different body fluids (blood, saliva, feces, and urine) converging on the role of biogenic amine metabolism in ASD, to the best of our knowledge, there are no studies integrating these multi-omics data. Much work remains to be carried out to identify and validate multiple biomarkers using standardized methodologies in multi-omics studies to improve diagnosis and to unravel the complex molecular mechanisms underlying ASD. However, the integration of these data remains a significant challenge, particularly when it comes to the subcategorization of ASD diagnoses based on individual genetic, neurobiological, and behavioral profiles. Most studies that have been carried out did not account for ASD heterogeneity. Future studies should stratify the ASD population according to their biological characteristics and search for specific biogenic amine genetic defects only in individuals with deregulated amine metabolic pathways. Such stratification will help to increase the molecular diagnostic yield.

## 8. Conclusions

In the study of BAs in relation to ASD, it is essential to consider other neurodevelopmental disorders and co-occurring conditions, as these often share overlapping neurobiological pathways. BAs, including serotonin, dopamine, and norepinephrine, are integral to neurophysiological processes that begin early in neurodevelopment, influencing key events such as neuronal differentiation, synaptogenesis, axonal guidance, and the modulation of neurotransmitter systems [215]. These processes are crucial for the proper formation and organization of neural circuits, which in turn govern behavioral regulation, social cognition, emotional processing, and executive functions. Dysregulation of these mechanisms during neurodevelopment has been implicated in ASD, contributing to the altered brain structures and functions often observed in individuals with ASD.

The complexity of these processes further complicates the identification of specific biomarkers and therapeutic targets for ASD. Despite these challenges, further research into the role of BAs in ASD offers considerable potential for advancing our understanding of the disorder. A deeper comprehension of how imbalances in these neurotransmitters contribute to the broad spectrum of ASD symptoms could ultimately lead to more targeted and effective diagnostic and treatment strategies, offering better outcomes for individuals with autism spectrum disorder. Clinicians must remain well informed about these developments, as advances in this area may one day enable more neurobiologically informed interventions, ultimately leading to personalized treatment approaches that are more effective.

## Figures and Tables

**Table 1:** Classification of biogenic amines.

Name	IUPAC Name	Classification	Chemical Structure
Serotonin	3-(2-aminoethyl)-1H-indol-5-ol	Monoamine	Heterocyclic
Histamine	4-(2-aminoethyl)benzene-1,2-diol	Monoamine	Heterocyclic
Dopamine	4-(2-aminoethyl)benzene-1,2-diol	Monoamine	Aromatic
Epinephrine	4-[(1R)-1-hydroxy-2-(methylamino)ethyl]benzene-1,2-diol	Monoamine	Aromatic
Norepinephrine	4-[(1R)-2-amino-1-hydroxyethyl]benzene-1,2-diol	Monoamine	Aromatic
Putrescine	butane-1,4-diamine	Diamine	Aliphatic
Spermidine	(4-aminobutyl)(3-aminopropyl)amine	Polyamine	Aliphatic
Spermine	(3-aminopropyl)({4-[(3-aminopropyl)amino]butyl})amine	Polyamine	Aliphatic

**Table 2:** Enzymes of serotonin (5-HT) metabolism and relative encoding genes. The association with autism spectrum disorder (ASD) is reported.

Enzyme Symbol	Name	Protein ID	E.C. Number	Gene Name	Gene ID	OMIM Gene ID	Disease Association (MIM Phenotype)	Gene Association with ASD (SFARI and Pubmed)	ASD or ASD Associated NDD SNV or CNV	Type of Study	Ref.
TPH	Tryptophan 5-monooxygenase; L-tryptophan 5-hydroxyilase (2 isoforme)	P17752	1.14.16.4	*TPH1*	7166	*191060	No association	Not listed in SFARI database; found on PubMed in association with autism	No association with autism	Genotyping	[49]
		Q8IWU9	1.14.16.4	*TPH2*	121278	*607478	Susceptibility to attention deficit-hyperactivity disorder-7 (#613003) and major depressive disorders (#608516)	Not listed in SFARI database; found on PubMed in association with autism	No association with autism	Genotyping	[49]
									Weak association of rs4341581 and rs11179000 with autism	Sanger sequencing	[50]
AADC	Aromatic-L-acid decarboxylase; dopa decarboxylase	P20711	4.1.1.28	*DDC*	1644	*107930	Aromatic-L-acid decarboxylase deficiency—AADCD (#608643)	Strong candidate gene	Association of rs6592961 with autism	Case-control	[51]
									c.1040 G > A (p.Arg347Gln)	WES and WGS	[52]
									c.1066_1068del (p.Arg356del)	WES	[53]
									c.1234 C > T (p.Arg412Trp)	WES and WGS	[54]
									c.1331 T > C (p.Phe444Ser)	WES	[55]
									c.480del (p.Thr161ProfsTer3)	WGS	[56]
									c.759 T > A (p.Asn253Lys)	Meta-analysis	[57]
									c.849 G > C (p.Glu283Asp)	WES	[53]
MAO (MAO-A)	Monoamine oxidase (2 isoforms)	P21397	1.4.3.4	*MAOA*	4128	*309850	Brunner syndrome (#300615)	Strong candidate gene	Association of rs6323 with autism (males only)	Case-control	[58]
									MAOA VNTR polymorphism association with autism	Case-control	[59,60,61]
									CNV, del chrXp11.3 (800kb)	Array-CGH	[62]
									CNV, del chrXp11.3-p11.4 (240 Kb)	Array-CGH	[63]
									c.133C > T (p.Arg45Trp)	WES	[64]
									c.1438-2A > G	WES and transcriptome profiling	[65]
									c.208G > A (p.Val70Met)	Targeted gene panel	[66]
									c.617G > A (p.Arg206Gln)	Targeted gene panel	[67]
									c.710A > T (p.Gln237Leu)	WGS	[68]
									c.730G > A (p.Val244Ile)	WES	[69]
									c.749_750insT (p.Ser251LysfsTer2)	WES	[64]
									c.797_798delinsTT (p.Cys266Phe)	Targeted gene panel	[70]
									c.815C > T (p.Ala272Val)	WES	[55]
									c.886C > T (p.Gln296Ter)	Sanger sequencing	[71]
AANAT (SNAT)	Serotonin N-acetyltransferase; arylalkylamine N-acetyltransferase	Q16613	2.3.1.87	*AANAT*	15	*600950	Not correlated with a MIM phenototype	Not listed in SFARI database; not found on PubMed in association with autism	/	/	/
ASMT (HIOMT)	Acetylserotonin O-methyltransferase; hydroxyindole O-methyltransferase	P46597	2.1.1.4	*ASMT*	438	*402500	Not correlated with a MIM phenototype	Strong candidate gene (2)	Association of rs4446909 with autism	Genotyping	[72]
									Association of rs5989681 with autism	Genotyping	[72]
									Association of several SNPs with autism	Genotyping	[73]
									CNV. delchrX	Array-CGH	[74]
									c.855 G > A (p.Trp257Ter)	Sanger sequencing	[75]
									c.-56C > A upstream variant	Sanger sequencing	[75]
									c.241A > G (p.Lys81Glu)	Sanger sequencing	[72]
									c.343C > T (p.Arg115Trp)	Sanger sequencing	[75]
									c.496G > A (p.Val166Ile)	Sanger sequencing	[75]
									c.51C > A (p.Asn17Ile)	Sanger sequencing	[72]
									c.536T > G (p.Val179Gly)	Sanger sequencing	[75]
									c.569G > A (p.Trp190Ter)	WES	[55]
									c.615G > A (p.Gln205=)	Sanger sequencing	[72]
									c.675C > A (p.Cys225Ter)	WGS	[56]
									c.917G > C (Gly306Ala)	Sanger sequencing	[72]
									c.976C > T (p.Leu326Phe)	Sanger sequencing	[72]
									IVS2 + 943T	Sanger sequencing	[75]
									IVS5 + 2T > C	genotyping	[72]
									IVS5 + 43G > C	Sanger sequencing	[75]

Genes were first searched for on the SFARI gene database of the Simons Foundation (https://gene.sfari.org/; accessed on 3 February 2025), and if not found, they were searched for on PubMed by entering the following keywords: “name of the gene AND autism.” For each gene, common and rare variants are reported in the table. Gene variants include SNVs (single-nucleotide variants) and CNVs (copy number variants). WES—whole-exome sequencing; WGS—whole-genome sequencing.

**Table 4:** Enzymes of catecholamine metabolism and relative encoding genes. The association with autism spectrum disorder (ASD) is reported.

Enzyme Symbol	Name	Protein ID	E.C. Number	Gene Name	Gene ID	OMIM Gene ID	Disease Association (MIM Phenotype)	Gene Association with ASD (SFARI and Pubmed)	ASD or ASD Associated NDD SNV or CNV	Type of Study	Ref.
TH	Tyrosine hydroxylase; tyrosine 3-monooxygenase	P07101	1.14.16.2	*TH*	7054	*191290	Segawa syndrome, autosomal recessive (#605407)	Not listed in SFARI database; not found on PubMed in association with autism	/	/	/
AADC	Aromatic-L-acid decarboxylase; dopa decarboxylase	P20711	4.1.1.28	*DDC*	1644	*107930	See above	See above	See above	See above	See above
MAO (MAO-A)	Monoamine oxidase	P21397	1.4.3.4	*MAOA*	4128	*309850	See above	See above	See above	See above	See above
COMT	Catechol-O-methyltransferase	P21964	2.1.1.6	*COMT*	1312	*116790	Panic disorder 1—PAND1(#167870) and schizophrenia—SCZD (#181500)	Not listed in SFARI database; found on PubMed in association with autism	rs4680 associated with increasing autism severity	Sanger sequencing	[145]
									CNV,delchr22q11.2	Array-CGH	[146,147]
DBH	Dopamine beta-hydroxylase; dopamine beta-monooxygenase	P09172	1.14.17.1	*DBH*	1621	*609312	Othostatic hypotension 1—ORTHYP1 (#223360)	Not listed in SFARI database; found on PubMed in association with autism	19-bp insertion/deletion polymorphism association with autism	PCR and gel electrophoresis	[148]
									c.910 G > T association with IQ in ASD	PCR and gel electrophoresis	[149]
PNMT	Phenylethanolamine N-methyltransferase	P11086	2.1.1.28	*PNMT*	5409	*171190	Not correlated with a MIM phenotype	Not listed in SFARI database; not found on PubMed in association with autism	/	/	/

**Table 5:** Enzymes of diamine/polyamine metabolism and relative encoding genes. The association with autism spectrum disorder (ASD) is reported.

Enzyme Symbol	Name	Protein ID	E.C. Number	Gene Name	Gene ID	OMIM Gene ID	Disease Association (MIM Phenotype)	Gene Association with ASD (SFARI and Pubmed)	ASD or ASD Associated NDD SNV or CNV	Type of Study	Ref.
ODC	Ornithine decarboxylase	P11926	4.1.1.17	*ODC1*	4953	*165640	Bachman-Bupp syndrome—BABS (#619075)	Not listed in SFARI database; found on PubMed in association with neurodevelopmental disorders	Preliminary evidence of rs138359527 association with neurodevelopment	In silico analysis	[190]
SAMDC	Adenosylmethionine decarboxylase 1	P17707	4.1.1.50	*AMD1*	262	*180980	Not correlated with a MIM phenotype	Not listed in SFARI database; not found on PubMed in association with autism	/	/	/
SRM	Spermidine synthase	P19623	2.5.1.16	*SRM*	6723	*182891	Not correlated with a MIM phenotype	Not listed in SFARI database; not found on PubMed in association with autism	/	/	/
SMS	Spermine synthase	P52788	2.5.1.22	*SMS*	6611	*300105	Intellectual developmental disorder, X-linked, syndromic, Snyder-Robinson type—MRXSSR (#309583)	Not listed in SFARI database; found on PubMed in association with autism	c.424C > T (p.Leu142Phe)	WES	[117]
SSAT	Spermidine/spermine N1-acetyltransferase 1	P21673	2.3.1.57	*SAT1*	6303	*313020	Not correlated with a MIM phenotype	Not listed in SFARI database; not found on PubMed in association with autism	/	/	/
PAO	Polyamine oxidase; N(1)-acetylpolyamine oxidase	Q6QHF9	1.5.3.13	*PAOX*	196743	*615853	Not correlated with a MIM phenotype	Not listed in SFARI database; not found on PubMed in association with autism	/	/	/
SMO	Spermina oxidase; polyamine oxidase 1	Q9NWM0	1.5.3.16	*SMOX*	54498	*615854	Not correlated with a MIM phenotype	Not listed in SFARI database; not found on PubMed in association with autism	/	/	/
MAO (MAO-B)	Monoamine oxidase	P27338	1.4.3.4	*MAOB*	4129	*309860	See above	See above	See above	See above	See above
DAO	Diamine oxidase; amine oxidase copper-containing 1	P19801	1.4.3.22	*AOC1*	26	*104610	See above	See above	See above	See above	See above

## Data Availability

Not applicable.
